# Correlation between the presence of neutralizing antibodies against porcine circovirus 2 (PCV2) and protection against replication of the virus and development of PCV2-associated disease

**DOI:** 10.1186/1746-6148-2-6

**Published:** 2006-01-30

**Authors:** Peter Meerts, Gerald Misinzo, David Lefebvre, Jens Nielsen, Anette Bøtner, Charlotte S Kristensen, Hans J Nauwynck

**Affiliations:** 1 Laboratory of Virology, Faculty of Veterinary Medicine, Ghent University, Belgium; 2 Danish Institute for Food and Veterinary Research, Department of Virology, Lindholm, Denmark; 3 Danish Bacon and Meat Council, Denmark

## Abstract

**Background:**

In a previous study, it was demonstrated that high replication of Porcine circovirus 2 (PCV2) in a gnotobiotic pig was correlated with the absence of PCV2-neutralizing antibodies. The aim of the present study was to investigate if this correlation could also be found in SPF pigs in which PMWS was experimentally reproduced and in naturally PMWS-affected pigs.

**Results:**

When looking at the total anti-PCV2 antibody titres, PMWS-affected and healthy animals seroconverted at the same time point, and titres in PMWS-affected animals were only slightly lower compared to those in healthy animals. In healthy animals, the evolution of PCV2-neutralizing antibodies coincided with that of total antibodies. In PMWS-affected animals, neutralizing antibodies could either not be found (sera from field studies) or were detected in low titres between 7 and 14 DPI only (sera from experimentally inoculated SPF pigs). Differences were also found in the evolution of specific antibody isotypes titres against PCV2. In healthy pigs, IgM antibodies persisted until the end of the study, whereas in PMWS-affected pigs they quickly decreased or remained present at low titres. The mean titres of other antibody isotypes (IgG1, IgG2 and IgA), were slightly lower in PMWS-affected pigs compared to their healthy group mates at the end of each study.

**Conclusion:**

This study describes important differences in the development of the humoral immune response between pigs that get subclinically infected with PCV2 and pigs that experience a high level of PCV2-replication which in 3 of 4 experiments led to the development of PMWS. These observations may contribute to a better understanding of the pathogenesis of a PCV2-infection.

## Background

Porcine circovirus type 2 is a member of the *Circoviridae *family. This family contains small viruses with a circular single stranded DNA genome, including a number of avian and mammal viruses that are known to cause persistent infections in their host. Porcine circovirus 2 (PCV2) and chicken anaemia virus are able to maintain a high level of replication up to one month or longer after experimental inoculation (Bolin *et al*., 2001; Imai *et al*., 1999) [1,2]. The human hepatitis-related TT-virus, also a member of the *circoviridae *family (Nishizawa *et al*., 1997) [3], is able to maintain a constant viremia for a period of more than 3 years in infected patients (Lefrere *et al*., 2000) [4]. The ability of these small viruses to persist and replicate for such a long time in their host, indicates a failure of the host's immunologic response to efficiently recognize and/or remove virus particles or virus-infected cells from the body.

The economical importance of PCV2 lies in its association with the postweaning multisystemic wasting syndrome (PMWS) (Ellis *et al*., 1998) [5], a condition in weaned pigs observed for the first time in 1991 (Harding *et al*., 1996) [6]. The syndrome is characterized by clinical signs such as anaemia, jaundice and severe weight loss and histopathological lesions including lymphocyte depletion and infiltration of monocytes in lymphoid tissues. It is generally accepted that other factors, including other viral infections such as porcine parvovirus (Allan *et al*., 2000a) [7] or porcine reproductive and respiratory syndrome virus (Allan *et al*., 2000b) [8] or a stimulation of the immune system (Krakowka *et al*., 2001) [9], influence the clinical outcome of the infection. A high and persisting PCV2-replication level was found to be a crucial factor in the pathogenesis of the disease. The demonstration of an intense PCV2-replication was therefore incorporated in the case definition of PMWS in order to differentiate the disease from other chronic disorders leading to the manifestation of similar clinical symptoms (Segalés and Domingo, 2002) [10].

After experimental PCV2-inoculation, a high variation in virus replication level and duration has been described between pigs (Ladekjær-Mikkelsen *et al*., 2002; Sanchez *et al*., 2003) [11,12]. In a subsequent study, the evolution in PCV2-replication was monitored in individual gnotobiotic pigs (Meerts *et al*., 2005) [13]. During the first two weeks of the experiment, the evolution of the PCV2-titre in inguinal lymph nodes of inoculated pigs showed little variation. At later stages of the infection, two patterns developed. In most pigs, PCV2-titres decreased between 15 and 21 DPI. One of the PCV2-inoculated pigs, showed a dramatic increase in PCV2-replication in this period, resulting in a significantly higher level of PCV2-replication at 21 DPI. Similar observations were made in other studies (Ladekjær-Mikkelsen *et al*., 2002; Sanchez *et al*., 2003) [11,12]. From these observations, it could be concluded that not all pigs are equally able to deal with a PCV2-infection. When the relation between the onset of the adaptive immune response against PCV2 was monitored in these pigs, an interesting observation was made. Using a sensitive PCV2-neutralization assay, PCV2-neutralizing antibodies could be detected in all PCV2-inoculated pigs starting from 15 DPI, except in the pig with high PCV2-replication (Meerts *et al*., 2005) [13]. This study suggested a correlation between the absence or delayed production of PCV2-neutralizing antibodies and an increased level of replication of the virus in experimentally inoculated pigs. The latter study was performed in gnotobiotic piglets. Previously, it has been shown that the adaptive immune response against viral infections in gnotobiotic pigs which are raised in a sterile environment may deviate to some extent from the response of pigs in a conventional environment (Mehrazar *et al*., 1993; Butler *et al*., 2002; Souza *et al*., 2004) [14-16]. Therefore, the observation made in PCV2-inoculated gnotobiotic piglets should be confirmed in naturally infected and experimentally inoculated conventional pigs.

The aim of the present study was to investigate whether the lack of PCV2-neutralizing antibodies could also be found in naturally or experimentally reproduced PMWS-affected pigs and to characterize more in detail the humoral immune response against PCV2 in PMWS-affected and healthy animals.

## Results

### Total PCV2-specific and PCV2-neutralizing antibody titres in experimentally inoculated gnotobiotic pigs

**Figure 1:**
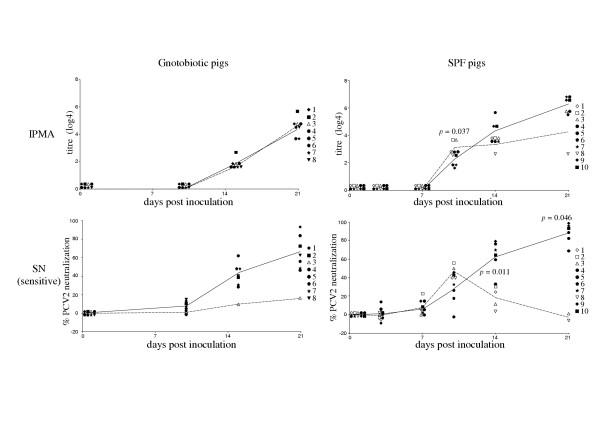
**Course of IPMA and SN (sensitive) antibody titres in experimentally PCV2-inoculated gnotobiotic and SPF pigs**. Full symbols: pigs with low PCV2 replication (gnotobiotic) or subclinically infected pigs (SPF); empty symbol: pig with high PCV2-replication (gnotobiotic) or PMWS-affected pigs (SPF).

The total anti-PCV2 antibody profiles in experimentally inoculated gnotobiotic and SPF pigs are shown in Figure 1. In this figure, results of the gnotobiotic pigs were presented in two groups based on the virological outcome of the infection. In gnotobiotic pigs no antibodies were detected before 15 DPI. Antibody titres increased in a time-dependent manner until the end of the study.

With the Classical SN, no PCV2-neutralizing antibodies were detected in any pig until the end of the study at 21 DPI. Therefore, the sensitive SN was applied. With this assay, PCV2-neutralizing antibodies were detected in gnotobiotic pigs starting from the same time point when anti-PCV2 antibodies were detected with the IPMA (15 DPI). In seven gnotobiotic pigs (pigs with low PCV2-replication) the evolution of PCV2-neutralizing antibody titres followed the evolution of the total anti-PCV2 antibody titre. However, one gnotobiotic pig (the pig with high PCV2-replication) showed a PCV2-neutralizing antibody profile that significantly differed from its total anti-PCV2 antibody pattern and from the neutralizing antibody pattern observed in the other pigs. In this pig no significant PCV2-neutralizing antibodies could be detected using the sensitive SN at any time during the study.

### Total PCV2-specific and PCV2-neutralizing antibody titres in experimentally inoculated conventional SPF pigs

The total anti-PCV2 antibody profiles in experimentally inoculated conventional SPF pigs are shown in Figure 1. In this figure, results of these pigs were presented in two groups based on the clinical outcome of the infection. In SPF pigs the first anti-PCV2 antibodies were detected at 10 DPI. At that time PMWS-affected SPF pigs showed significantly higher IPMA titres compared to their healthy group mates (*p *= 0.037). Only in one PMWS-affected SPF pig (pig 3), a plateau was reached starting from 10 DPI. At 21 DPI, the antibody titre in this pig was low (2.7 log4) compared to the other pigs (5.7 – 6.7 log4).

With the Classical SN, similar as in gnotobiotic pigs, no PCV2-neutralizing antibodies were detected until the end of the study thus the sensitive SN was applied. With this assay, PCV2-neutralizing antibodies were detected starting from 10 DPI.

In six SPF pigs (pigs that did not develop PMWS) the evolution in PCV2-neutralizing antibody titres followed the evolution of the total anti-PCV2 antibody titre. In these pigs, the PCV2-neutralizing antibody titres increased in a time dependent manner until the end of the study. At the end of the study (21 dpi) up to 98% PCV2-neutralization was observed in some pigs. The four SPF pigs that developed PMWS showed a PCV2-neutralizing antibody profile that significantly differed from their total anti-PCV2 antibody pattern and from the neutralizing antibody pattern observed in the other pigs in the experiment. These pigs showed a small peak in PCV2-neutralization at 10 DPI (40 to 56%) but afterwards the neutralization titres declined towards the time point at which the last serum samples could be collected (two pigs died at 20 DPI) or until neutralization could no longer be detected (2 pigs at 21 DPI). SN-titres in PMWS-affected SPF pigs were significantly lower compared to SN titres in healthy pigs at 14 (*p *= 0.011) and 21 DPI (*p *= 0.046).

### Total PCV2-specific and PCV2-neutralizing antibody titres in naturally PCV2-infected pigs

**Figure 2:**
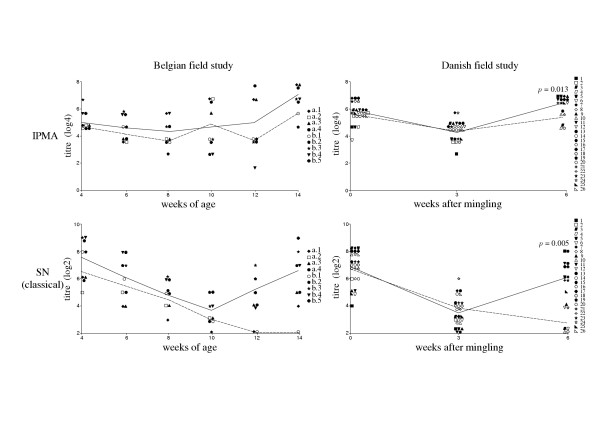
**Course of IPMA and SN (Classical) antibody titres in naturally PCV2-infected pigs in Belgian and Danish field conditions**. Full symbols: subclinically infected pigs; empty symbols: PMWS-affected pigs. Full line: mean antibody titre of subclinically infected pigs; dashed line: mean antibody titre of PMWS-affected pigs. When difference between mean titres was significant, the *p*-value was shown.

Total PCV2-specific antibody titres (IPMA) and PCV2-neutralizing titres (classical SN) detected in naturally PCV2-infected pigs derived from a Belgian and a Danish field study are presented in Figure 2. In this figure, results of pigs were presented in two groups based on the clinical outcome of the infection. All piglets enclosed in both field studies showed maternally derived antibody (MDA) titres between 3.7 and 6.7 log_4_. A gradual decline of total anti-PCV2 antibody titres was detected in all pigs except one pig (pig 6) in the Danish study. Seroconversion was observed in the Danish pigs between 3 and 6 weeks after mingling and in the Belgian pigs between 6 and 12 weeks of age. In the Danish study, IPMA antibody titres at 6 weeks post mingling were generally lower in PMWS-affected pigs, compared to healthy pigs (*p *= 0.013). However, this difference could not be demonstrated in the Belgian field study.

At the onset of both field studies, SN titres were found above the detection limit with the classical SN in all sera due to the presence of MDA. SN titres at the start of the studies ranged between 4 and 9. At the start of the study, no significant differences in SN Ab titres could be observed between subclinically infected pigs and pigs that developed PMWS. SN titres decreased in a time-dependent manner during the first weeks of the study. In all but one subclinically infected pigs in the Danish field study, a rise in SN titres could be detected before the end of the study. Such a seroconversion was detected starting from 10 weeks of age in the Belgian study and starting from 3 weeks after mingling in the Danish study. The majority of the PMWS affected pigs died before they reached the age at which a rise in SN titres could be detected (1 pig in the Belgian and 8 pigs in the Danish study). However, one PMWS-affected pig in the Belgian study and 5 pigs in the Danish study survived until the end of the study. In none of these pigs, a rise in SN titres was detected. This resulted in a significant lower SN-titre in the PMWS affected pigs in the Danish study (*p *= 0.005).

### Course of PCV2-specific antibody isotype titres in experimentally inoculated gnotobiotic and SPF pigs

**Figure 3:**
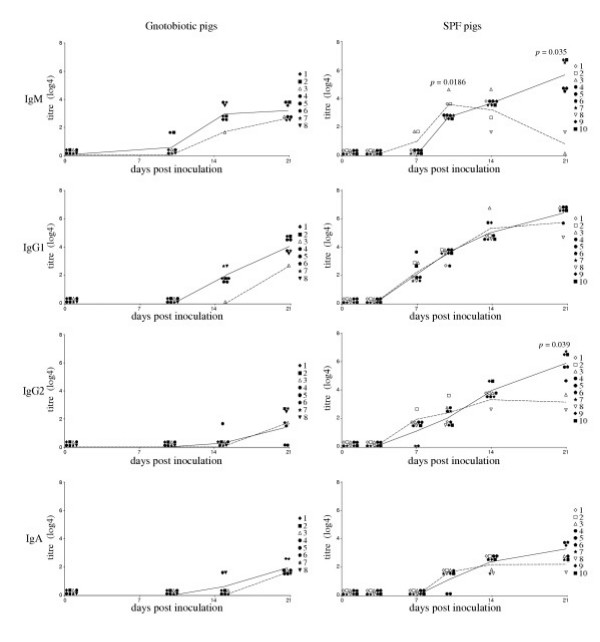
**Course of isotype-specific antibody titres in experimentally PCV2-inoculated gnotobiotic and SPF pigs**. Full symbols: pigs with low PCV2 replication (gnotobiotic) or subclinically infected pigs (SPF); empty symbol: pig with high PCV2-replication (gnotobiotic) or PMWS-affected pigs (SPF). Full line: mean antibody titre of the pigs with low PCV2 replication (gnotobiotic) or healthy pigs (SPF). Dashed line: antibody titre of pig with high PCV2-replication (gnotobiotic) or mean titre of PMWS-affected pigs (SPF). When the difference between the mean titres was significant, the *p*-value was shown.

The titres of specific anti-PCV2 antibody isotypes in gnotobiotic and SPF pigs are shown in Figure 3. In gnotobiotic pigs, the different antibody isotypes generally followed the course of the IPMA antibody titres. The IgM, IgG1 and IgG2 titres detected in the pig that experienced a high level of PCV2-replication (gnotobiotic pig 3) did not differ from the titres of the other pigs. In the pig with high PCV2-replication IgA-type antibodies were detected starting from 15 DPI whereas in the other pigs IgA-type antibodies were detected starting from 10 DPI.

In SPF pigs, a clear difference between PMWS-affected and healthy pigs was observed in the evolution of IgM-type antibodies. Between healthy pigs, very little variation was detected in IgM titres. Starting from 10 DPI, anti-PCV2 IgM-antibodies were detected and titres steadily increased until the end of the study. In 2 out of 4 PMWS-affected pigs, IgM antibodies were detected starting from 7 DPI. At 10 DPI, IgM titres in PMWS-affected pigs were significantly higher than IgM titres in healthy pigs (*p *= 0.018). After 10 DPI, IgM titres in PMWS-affected pigs decreased towards the end of the study. At 21 DPI, IgM titres in PMWS-affected pigs were significantly lower compared to IgM titres in healthy pigs (*p *= 0.035).

IgG1, IgG2 and IgA titres in SPF pigs generally followed the profiles of the IPMA antibody titres. Titres increased until the end of the study. At 21 DPI, the mean titres of these antibody isotypes in PWMS-affected pigs were lower compared to the titres in healthy pigs. However, only for the IgG2 titres, these differences were found to be significant (*p *= 0.039).

### Course of PCV2-specific antibody isotype titres in naturally PCV2-infected pigs

In naturally PCV2-infected pigs, similar observations were made as in the experimentally inoculated animals. In both field studies, the course of the IgM-type antibodies against PCV2 differed between healthy and PMWS-affected pigs. In healthy pigs, IgM titres were found to increase steadily towards the end of the study, resulting in high IgM titres even at 14 weeks of age, 6 weeks after the first IgM antibodies were detected in some pigs. PMWS-affected pigs had low titres of IgM antibodies. The mean IgM titres in PMWS-affected pigs in the Danish study were significantly lower compared to the titres in healthy pigs (*p *= 0.001). IgG1, IgG2 and IgA titres followed the course of the IPMA antibody titres. It was observed that the mean of these titres in PMWS-affected pigs was always lower compared to the mean titre in healthy pigs. However, these differences were not significant. Due to the limited number of PMWS-affected pigs in the Belgian field study, it was not possible to detect significant differences but it was clear that also in this study IgM titres in the affected pigs were much lower compared to titres in their healthy littermates.

## Discussion

In the present study, it was shown that the absence of PCV2-neutralizing antibodies was correlated with high PCV2-replication and with PCV2-related disease (PMWS) if present.

Neutralizing antibodies have important functions in the defence of animals and humans against viral infections. Starting from the moment they appear, neutralizing antibodies bind and neutralize the virus and prevent further spread in the body by inhibiting attachment and infection of new cells. It has been shown that neutralizing antibodies against many viruses are more rapidly induced during the immune response and reach higher titres compared to antibodies against other viral epitopes (Bachmann and Zinkernagel, 1997) [26]. In contrast, neutralizing antibodies against PCV2 could only be detected starting from 10 dpi by using a particularly sensitive SN assay (Meerts *et al*., 2005) [13]. These SN titres rose slowly until they could be detected with the classical SN assay starting from 4 weeks after inoculation (Pogranichnyy *et al*., 2000) [24]. The slow induction of neutralizing antibodies is a very effective mechanism for the virus to ensure a long period of replication in its host. In previous work, the observation was made that an increased replication of the virus in one pig, was correlated with a lack of neutralizing antibody production (Meerts *et al*., 2005) [13]. In the present study, it was confirmed that the absence of PCV2-neutralizing antibodies is indeed a recurring phenomenon in pigs with high PCV2-replication. In experimentally PCV2-inoculated SPF pigs and in two field studies, a good correlation was observed between the absence or low titres of neutralizing antibodies against PCV2 and the occurrence of a high virus replication and the development of PMWS. This observation reveals a possible mechanism that allows the virus to replicate to a higher extent in a limited number of pigs. These pigs seem to be unable to recognize or produce antibodies against the epitope(s) that is (are) involved in the neutralization of the virus. With relation to other viral infections such as pseudorabies virus (Jacobs and Kimman, 1994) [27], influenza virus (Lambkin and Dimmock, 1995) [28] or human rotavirus (Green *et al*., 1990) [29], it has been shown that different animals do not all recognize the same epitopes. Various syndromes have already been ascribed to the inability of certain individuals to recognize certain epitopes (susceptibility to mastitis (Lunden *et al*., 1990) [30], clinical outcome of Marek's disease virus (Wakenell *et al*., 1996) [31]). The susceptibility to these syndromes is highly genetically determined. It has been demonstrated previously that the prevalence of PMWS highly differs between regions. Since contemporary pig industry still uses region-specific breeds, this could be interpreted as an indication that PMWS would be partly genetically determined. Therefore, it would be worthwhile to check for a similar mechanism in the recognition of PCV2-neutralizing epitope and the subsequent influence on the susceptibility of the host to PMWS.

In the present study, it was shown that maternally derived antibodies in serum were able to neutralize PCV2-infection in an *in vitro *SN assay but could not avoid PCV2-infection of the pig since seroconversion was observed in pigs with maternal SN titres as high as 6 log2. When looking at the total antibody titres (IPMA) in the field studies, it was observed that seroconversion against PCV2 never occurred in pigs before weaning at the age of 4 weeks (Belgian field study) or before the pigs were mingled (Danish study).

In experimentally inoculated SPF pigs that developed PMWS, a small and transient peak in PCV2-neutralization was observed at 10 DPI. It was observed that these pigs also showed a peak of IgM titres at that same time point. The IgM antibodies in these pigs did not lead to the production of neutralizing IgG or IgA antibodies, which may indicate that the neutralizing epitope was not targeted by the humoral immune response. However, the pronounced B-cell lymphopenia that was observed in these pigs (Nielsen *et al*., 2003) [32] may also constitute an important factor in the mechanism causing these changes in the humoral immune response. The parallel evolution of the SN and IgM titres in these pigs suggests that the IgM antibodies might be responsible for the low level of PCV2-neutralization that was observed at 10 DPI. Possibly IgM antibodies which are directed against non-neutralizing epitopes, are able to neutralize PCV2 to some extent by sterical hindrance. Indeed, IgM antibodies are secreted in a pentamere formation that is much larger compared to any other antibody isotype. This pentamere form contains 10 Fab fragments that would enable it to bind PCV2 at different sites. In PMWS-affected pigs in the Danish field study, a peak in IgM antibodies was not detected probably because time intervals between the different blood collections were too long (3 weeks). When comparing IgM profiles in both experimental studies, it is observed that in the gnotobiotic pig with high PCV2-replication, low IgM titres were detected until the end of the study. In general the humoral immune response in gnotobiotic pigs was delayed compared to the SPF pigs, which might be the reason that no decrease in IgM titres was observed in this pig. The low IgM titres in this gnotobiotic pig might correspond to the slight increase in PCV2-neutralization (up to 16% whereas in other pigs they reached 97%) that was observed in this pig. This slight neutralization however, was not significantly different compared to the result obtained from serum of the same pig before inoculation.

In contrast with titres in PMWS-affected pigs, IgM titres in subclinically infected animals persisted and even increased until the end of the study. After a viral infection, IgM-type antibodies are the first antibodies that are being produced. Generally, the plasma cells that produce these antibodies go through an isotype switch and start producing other isotypes of antibodies resulting a down-regulation of IgM production (Esser and Radbruch, 1997) [33]. A persistence of IgM type antibodies has already been described during the immune response against other viruses such as the coronavirus responsible for the severe acute respiratory syndrome in humans (Woo *et al*., 2004) [34] or the human parvovirus B19 (Yeagashi *et al*., 1989) [35]. However, the mechanism of this prolonged presence of IgM antibodies has not been defined yet. It is known that in older animals such as breeding sows, exceptionally high IPMA antibody titres against PCV2 can be found compared to antibody titres against other viruses. It may be hypothesized that the persistent presence of IgM lies at the base of this observation.

Although a clear difference in the course of IgM antibodies was observed between healthy and PMWS-affected pigs, the isotype switch occurred in both kinds of pigs at the same time point since other antibody isotypes were detected starting from the same time point. In PMWS-affected pigs, titres of other antibody isotypes against PCV2 were somewhat lower compared to titres in healthy pigs at the end of the studies. These differences, although hard to explain, indicate that some important changes in the humoral immune response occur. Possibly they are related to the fact that PCV2 is able to cause a severe depletion of especially the B-cells (Sanchez *et al*., 2004; Nielsen *et al*., 2003) [32,36]. Whether these changes are important in the induction of PMWS or are merely a consequence of it, remains unclear.

## Methods

### Sera

In the present study, sera from four different studies were included. In these studies, pigs got experimentally or naturally infected with PCV2. In three out of four studies PMWS was observed and diagnosed as proposed by Sorden (2000) [17] which implies the combined presence of the clinical symptoms and histopathological lesions associated with PMWS and the detection of PCV2 in these lesions. An overview of the used sera is shown in Table 1. A first set of sera was derived from two experimental PCV2-inoculation studies, one performed in 19-day-old gnotobiotic pigs and another performed in 21-day-old PCV2-negative conventional specific pathogen free (SPF) pigs. A second set of sera originated from two field studies performed in Belgium and Denmark.

1. Experimentally inoculated gnotobiotic pigs (Meerts *et al*., 2005) [13]: 8 gnotobiotic piglets were inoculated with PCV2 (10^4.3 ^TCID_50 _of strain 1121 intraperitoneally and oronasally) at 19 days of age. Blood was taken from these piglets at 0, 10, 15 and 21 days post inoculation (DPI). In this experiment, PMWS was not observed but one pig showed a high level of PCV2-replication at the end of the experiment (10^4.3 ^TCID_50_/g inguinal lymph node) compared to the other PCV2-inoculated pigs (average of 10^2.3 ^TCID_50_/g inguinal lymph node).

2. Experimentally inoculated SPF pigs (Ladekjær-Mikkelsen *et al*., 2002) [11]: 10 PCV2-negative SPF-pigs were inoculated with PCV2 (10^6.3 ^TCID_50 _of strain OSU3 intranasally) at the age of 3 weeks. At the time point of inoculation, 5 pigs were injected with keyhole limpet hemocyanin in incomplete Freunds adjuvant. However, this treatment could not be associated with any significant difference in clinical, immunological or virological outcome of the infection. Blood was taken at 0, 3, 7, 10, 14 and 21 DPI. Four out of 10 inoculated pigs developed PMWS and died or were euthanized before the end of the study (2 pigs at 20 DPI, 2 pigs at 21 DPI). These pigs showed high titres of PCV2 at the time they died (average of 10^5.1 ^TCID_50_/g inguinal and prescapular lymph node). At the end of the study, no PCV2 could be isolated from lymph nodes of pigs that did not develop PMWS.

3. Belgian field study (Meerts *et al*., 2004) [18]: during this study, blood was taken every two weeks from 9 piglets originating from 2 litters on PMWS-affected farms in Belgium. This serological follow-up started before the appearance of PMWS-related clinical signs (4 weeks of age) and ended at 14 weeks of age, the age after which new cases of PMWS seldom occur. In each litter, one piglet developed PMWS during the study and died before the end of the study.

4. Danish field study (Kristensen *et al*., 2004) [19]: in this field study, pigs originating from PMWS-affected and healthy farms were weaned and brought to the same unit. Blood was taken at the day the pigs were mingled (day 0) and 3 and 6 weeks later. During the study, a number of pigs from healthy farms developed PMWS and some of these affected pigs had to be euthanized before the end of the study. In the present study, sera were included originating from 13 subclinically infected pigs (healthy) and 13 pigs that developed PMWS during the follow-up. Eight affected pigs died before the last blood sampling occurred, five pigs developed PMWS but survived until the end of the study.

### Immunoperoxidase monolayer assay (IPMA)

The total titre of anti-PCV2 antibodies (IPMA-Ab) was determined using the IPMA as described previously by Labarque *et al*. (2000) [20]. This assay does not discriminate between neutralizing and non-neutralizing antibodies nor does it detect specific antibody isotypes.

### Isotype-specific IPMA

The individual levels of antibody isotypes IgG1, IgG2, IgA and IgM against PCV2 in sera were determined by an isotype specific IPMA. PCV2-infected PK-15 cell cultures were fixed and incubated with 4-fold dilution series of the tested sera. After 90 minutes of incubation, the cell cultures were washed thoroughly (4 times in phosphate buffered saline + 0,1% Tween 80) to remove unbound antibodies. Afterwards, mouse monoclonal antibodies directed against porcine IgM, IgA, IgG1 or IgG2 were added and incubated for 60 minutes (Labarque *et al*., 2000b) [21]. After additional washing, peroxidase-conjugated goat polyclonal antibodies against mouse immunoglobulins were added (Goat-anti-Mouse-HRP, DAKO) and peroxidase activity was visualized by adding 3-amino-5-ethylcarbazole.

### Classical seroneutralization assay (Classical SN)

With this assay, PCV2-neutralizing antibodies were detected using the classical protocol as has been described for many other viruses (Nauwynck *et al*., 1999; Van Reeth *et al*., 2003) [22,23]. Two-fold dilution series of the tested serum were prepared in MEM. The lowest dilution used in the test was 50% (1:1 dilution of serum in MEM). Twenty-five μl of these serial diluted sera were incubated for 1 hour at 37°C with a fixed number of infectious particle of PCV2 (10^3.7 ^TCID_50 _of strain Stoon-1010 in 25 μl). This mixture was afterwards added to 50% confluent PK-15 cells in a 96-well plate. After 1 hour of incubation at 37°C, the inoculum was removed, cell cultures were washed twice with MEM and culture medium was added. Inoculated cultures were further incubated at 37°C in an atmosphere containing 5% CO_2_. After 36 hours of incubation, cells were fixed and stained with the immunoperoxidase assay as described before. By light microscopy, it was observed at which concentration of the serum, PCV2-positive cells could still be detected. The neutralization titre with this assay was calculated as the reciprocal of the highest dilution of the serum that was able to completely block PCV2-infection in PK-15 cells. Thus the lowest dilution contained 25% serum (1:1 dilution of serum + equal volume of PCV2 stock), thereby the detection limit of this assay was 2 log_2_.

### Sensitive seroneutralization assay (sensitive SN)

It has previously been shown that PCV2-neutralizing antibody titres are only slowly induced (Pogranychnyy *et al*., 2000) [24]. Therefore, a more sensitive technique was developed. When no PCV2-neutralizing antibodies could be detected with the Classical SN, PCV2-neutralizing antibodies were detected by using the more sensitive seroneutralization described by Meerts *et al*. (2005) [13]. A similar neutralization assay had previously also been used to detect neutralizing antibodies against porcine reproductive and respiratory syndrome virus (Delputte *et al*., 2005) [25]. Briefly, a standard infectious dose of PCV2 (10^3.7 ^TCID_50 _of strain Stoon-1010) was incubated for 1 hour at 37°C with 25% of the serum. Afterwards, this virus-serum mixture was inoculated on PCV-negative PK-15 cells. After 1 hour, the inoculum was removed, cell cultures were washed 2 times, new cell medium was added and cultures were maintained at 37°C in the presence of 5% CO2. After 36 hours of incubation, cells were fixed and stained for PCV2 antigens using an immunoperoxidase technique. The number of PCV2-infected cells was counted and compared to the number of infected cells in a PK-15 culture inoculated with a PCV2 stock treated with the serum of the pig before inoculation. The result of this seroneutralization assay was expressed as a percentage of reduction of PCV2-infection.

### Statistical analysis

Statistical significance of differences was calculated using the Kruskal-Wallis test. Differences were considered significant when *p *< 0.05.

## Figures and Tables

**Figure 4:**
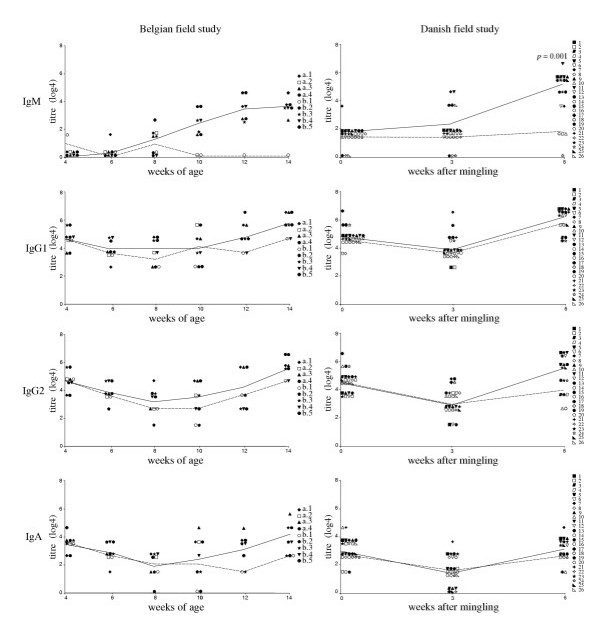
**Course of isotype-specific antibody titres in naturally PCV2-infected pigs**. Full symbols: subclinically infected pigs, empty symbols: PMWS-affected pigs. Full line: mean antibody titre of healthy pigs, dashed line: mean antibody titre of PMWS-affected pigs. When the difference between the mean values was significant, the *p*-value was shown.

**Table 1:** Sera used in the present study

			Number of pigs with	
			
Origin of pigs	PCV2-infection	Serum samples available	high* PCV2-replication	clinical signs of PMWS
Gnotobiotic	experimental	0, 10, 15, 21 DPI	1/8	0/8
SPF conventional	experimental	0, 7, 10, 14, 21 DPI	4/10	4/10
Field cases (Belgium)	natural	4, 6, 8, 10, 12, 14 weeks of age	NA**	2/10
Field cases (Denmark)	natural	0, 3, 6, 9 weeks after mingling	NA**	13/26

high*: = 10^4.3 ^TCID_50_/gram lymph node

NA**: not available since PCV2-titres were not obtained from healthy pigs. All PMWS-affected pigs did contain high titres of PCV2
